# Genomic surveillance reveals multiple origins and local transmission of travel-associated chikungunya virus in Yunnan, China

**DOI:** 10.1186/s12866-026-05100-w

**Published:** 2026-04-25

**Authors:** Mengyuan Zheng, Jie Li, Wei Chang, Hui Xiao, Li Liu, Weihong Qin, Xueshan Xia, Yue Feng

**Affiliations:** 1 Faculty of Life Science and Technology & Yunnan Provincial Key Laboratory of Public Health and Biosafety, Kunming University of Science and Technology, No. 727 Jingming Road, Chenggong Campus, Kunming, China; 2 Kunming Customs Port Outpatient Department, Yunnan International Travel Healthcare Center, Kunming, China; 3 Yunnan Provincial Key Laboratory of Public Health and Biosafety, Kunming Medical University, Kunming, Yunnan China

**Keywords:** Chikungunya virus, Yunnan, Phylogenetic analysis, Imported cases

## Abstract

**Background:**

Chikungunya virus (CHIKV) is a mosquito-borne arbovirus causing debilitating arthralgia, posing a significant public health threat in Southeast Asia. Bordering multiple endemic countries, Yunnan Province, China, is at high risk for CHIKV introduction.

**Methods:**

We conducted a retrospective analysis of 447 blood samples from febrile travelers arriving at Kunming Airport between May 2019 and January 2020. Viral detection was conducted by RT-qPCR, and near-complete genome sequences were subsequently obtained through RT-nested PCR and Sanger sequencing. To map genetic diversity and trace origins, we integrated 28 newly generated genomes with 66 publicly available sequences from local outbreaks and mosquitoes for phylogenetic and phylodynamic analysis.

**Results:**

Among the travelers, 32 (7.2%, 32/447) were CHIKV-positive, with 28 near-complete genomes successfully sequenced. Phylogenetic analysis revealed that all strains studied belonged to the Indian Ocean lineage (IOL) of the East/Central/South African (ECSA) genotype and contained the E1-211E, E1-226 A, and E2-264 A mutations, which are known to enhance *Aedes albopictus* transmission. By integrating publicly available GenBank records (*n* = 66), including local outbreaks (*n* = 56) and mosquito vector samples (*n* = 10), our comprehensive analysis identified three circulating genotypes in Yunnan: IOL, Asian, and West African. Phylodynamic analysis suggested that the IOL strains were closely related to sequences reported from Thailand, while the Asian genotype showed close genetic association with strains from Indonesia.

**Conclusions:**

The co-circulation of multiple genotypes, detected in air travelers, local cases, and mosquitoes, underscores the role of international travel in seeding CHIKV transmission in Yunnan. Our findings highlight the need for enhanced airport screening, genotype-aware surveillance, and coordinated vector control to mitigate this public health challenge in southwestern China.

**Supplementary Information:**

The online version contains supplementary material available at 10.1186/s12866-026-05100-w.

## Background

Chikungunya fever is an infectious disease caused by the Chikungunya virus (CHIKV), which is transmitted primarily by the bite of infected mosquitoes. Approximately 1 million people are infected annually, and outbreaks have increased significantly in recent years [1]. The geographic distribution of CHIKV has expanded substantially, particularly in tropical and subtropical regions, with large-scale outbreaks now occurring in areas where the disease had rarely been reported. According to the World Health Organization (WHO), between January 1 and September 30, 2025, a total of 445,271 suspected and confirmed cases and 155 deaths were reported from 40 countries [2]. The disease burden varied across regions. The Americas reported the largest number of cases (228,591 suspected infections), with Brazil accounting for approximately 96% of regional cases [2]. Large outbreaks were also reported in the European overseas territory of Réunion Island (54,517 confirmed cases) and in mainland Europe, where autochthonous transmission occurred in France (383 cases) and Italy (167 cases) [3, 4]. CHIKV remains actively transmitted in South and Southeast Asia, with over 34,000 suspected and confirmed cases reported, predominantly in India (32,617 cases), followed by Thailand (1,128 cases) [2]. Notably, mainland China also experienced a large-scale outbreak, with more than 10,000 confirmed cases reported between July and August [5].

Patients with chikungunya fever typically experience a sudden onset of high fever and severe joint pain, especially in the hands and feet, accompanied by rashes and muscle aches. Chronic joint pain may last for months or even years [6]. Although two vaccines against CHIKV have recently been approved, challenges related to safety, durability of immunity, and accessibility remain [7–9]. Moreover, there is no specific medication for chikungunya; treatment primarily aims to provide symptomatic relief. Thus, CHIKV infection remains a major focus of global public health research.

Recently, the risk of imported transmission of CHIKV has increased significantly due to global warming, increased trade, and travel. This risk is particularly pronounced in areas with high levels of travel and widespread mosquito populations [10]. Since 2008, when the first imported cases of chikungunya were reported in Guangdong Province, China has continued to report cases from abroad. Between 2010 and 2019, a total of 94 imported cases of chikungunya were documented in China, with 68 cases reported in 2019 alone. Most of these cases occurred between July and November and were mainly from Southeast Asian countries such as Myanmar, Thailand, Bangladesh, India, and the Philippines [11]. In addition, imported cases have led to four local outbreaks in China, primarily in the provinces of Yunnan, Guangdong, and Zhejiang [12]. As a result, imported CHIKV cases represent a significant global public health challenge. It is essential to strengthen surveillance, prevention, and control measures, and to promote vaccine development and immunization efforts to address the issue of imported transmission effectively.

Yunnan Province is located in the border region between China and neighboring countries such as Myanmar, Laos, and Vietnam. The frequent movement of people across these borders has resulted in many imported cases of CHIKV [13]. Additionally, Yunnan’s climate is conducive to the breeding and reproduction of Aedes *aegypti* and *Aedes albopictus*, thereby increasing the risk of CHIKV transmission and the likelihood of local outbreaks. In 2019, nearly 100 imported cases of chikungunya fever were reported in the Kunming, Ruili, and Gengma regions, the highest number of imported cases for the year [6]. Local outbreaks occurred in Ruili and Xishuangbanna, with 91 indigenous cases reported in Ruili [14, 15]. Notably, during the concurrent dengue epidemic in Xishuangbanna, surveillance of febrile patients identified 86 cases co-infected with CHIKV and dengue virus, indicating the co-circulation of these two arboviruses in the region [16]. In this study, we conducted a retrospective investigation of the prevalence and genetic diversity of 32 imported cases of chikungunya fever in Yunnan, China, from May 2019 to January 2020. We analyzed the genotypes and evolutionary relationships of CHIKV sequences from these cases and compared them with sequences available in the GenBank database, including those from local outbreaks and mosquitoes in Yunnan, China.

## Methods

### Study participants

From May 1, 2019, to January 30, 2020, all incoming international travelers at Kunming Changshui International Airport were screened for fever upon entry. Travelers with body temperature ≥ 37.0 °C were classified as febrile, and blood samples were collected from them. The inclusion criteria were: (i) available blood samples for testing; (ii) complete demographic and clinical data, including age, sex, nationality, importing country (the country visited during the potential incubation period), and clinical symptoms (headache, myalgia, arthralgia, rash, cough, chills, vomiting, and diarrhea). The exclusion criterion was: (i) individuals who had visited two or more countries during the potential incubation period. A total of 447 individuals met the inclusion criteria and were included in the study. Whole blood samples were collected in K3-EDTA tubes, and plasma was separated and stored at − 80 °C for RNA extraction. This study was conducted in accordance with the Declaration of Helsinki and approved by the Medical Ethics Committee of Kunming University of Science and Technology (Approval No: KUST-MEC-097).

### RNA extraction, CHIKV full-length genome amplification, and sequencing

Viral RNA was extracted from 200 µL of plasma using a MiniBEST Viral RNA/DNA Extraction Kit (TIANGEN, Beijing, China) according to the instructions in the kit manual. Multiplexed one-step qualitative real-time RT-PCR was performed to amplify gene fragments using the ZIKV/DENV/CHIKV Realtime PCR Kit (KECAN, Kunming, China). The test results showed that 32 samples were positive for CHIKV RNA. The full-length genome was amplified using 12 overlapping RT-PCR fragments, as previously described [17]. Primer sequences are listed in Table S1. Each amplicon was approximately 1,100 bp in length, with overlaps of ~ 140 bp between adjacent fragments (Fig. S1). To improve amplification sensitivity and specificity, a nested PCR strategy was employed. The first round of amplification was performed using a one-step RT-PCR kit (Vazyme), followed by a second round of nested PCR using inner primers. PCR reactions were carried out according to the manufacturer’s instructions. PCR products were visualized on a 1.0% agarose gel and purified using a DNA purification kit. Among the 32 CHIKV-positive samples, 28 successfully yielded all 12 expected amplicons and were subjected to subsequent sequencing analysis. Purified PCR products were sequenced in both directions using an ABI 3730XL DNA Analyzer (TSINGKE, Beijing, China). Sequence assembly was performed using SeqMan software (DNASTAR, Inc., Madison, WI, USA). Raw chromatograms were manually inspected, and sequences with overlapping peaks or low-quality reads were excluded. Overlapping regions between adjacent fragments were compared to ensure sequence consistency, and at least two independent reads supported each nucleotide position. The final consensus sequences spanned nucleotides 77 to 11,313 of the reference strain LR2006_OPY1 (GenBank accession no. DQ443544), resulting in a sequence of 11,237 bp covering the complete coding region. No gaps or ambiguous bases were observed, indicating high sequence accuracy and completeness.

### Phylogenetic analysis

NCBI BLASTn was used to identify the strain most closely related to our samples, and representative sequences were retrieved for phylogenetic analysis. Multiple sequence alignments were performed using the online version of MAFFT (v7.310) with the default parameters. A maximum likelihood (ML) phylogeny was constructed from this dataset. The phylogenetic model and tree inference were performed simultaneously using IQ-TREE v2.0.3, with 1000 ultrafast bootstrap replicates. The GTR + I + G model was determined to be the best-fitting nucleotide substitution model for the dataset.

### Bayesian MCMC evolutionary analyses

The Bayesian coalescent analysis was executed using the Markov Chain Monte Carlo (MCMC) sampling method in BEAST v1.8.3. The present analysis employed an uncorrelated lognormal relaxed molecular clock in conjunction with the General Time Reversible nucleotide substitution model, which incorporates a gamma-distributed rate and invariable sites (GTR + G4 + I). A Bayesian skyline coalescent tree prior was used for all analyses. The mean substitution rate (ucld.mean) was estimated from the data, and rate variation among branches was modeled using a lognormal distribution. This study analyzes two datasets: the CHIKV ECSA-IOL clade dataset and the Asian genotype dataset. For the ECSA-IOL clade, three independent MCMC chains were executed, with each chain undergoing 200 million steps. In contrast, two chains of 50 million steps were executed for the Asian genotype. In all instances, parameters were sampled at an interval of 1,000 steps. The initial 10% of the samples were discarded as burn-in, and the remaining chains were combined using LogCombiner. Convergence and effective sample sizes (ESS > 200) for all parameters were evaluated using Tracer v1.7.2 to ensure adequate mixing and reliable parameter estimation. Subsequently, a maximum clade credibility (MCC) tree was generated in TreeAnnotator, employing median node heights and 95% highest posterior density (HPD) intervals for visualization purposes. The tree was subsequently exported and processed using FigTree v1.4.4. The specific operational procedures, along with parameter selection and configuration for the analysis software, were conducted in accordance with previous reports [18].

## Results

### Epidemiologic characteristics of patients with CHIKV infection

**Fig. 1:**
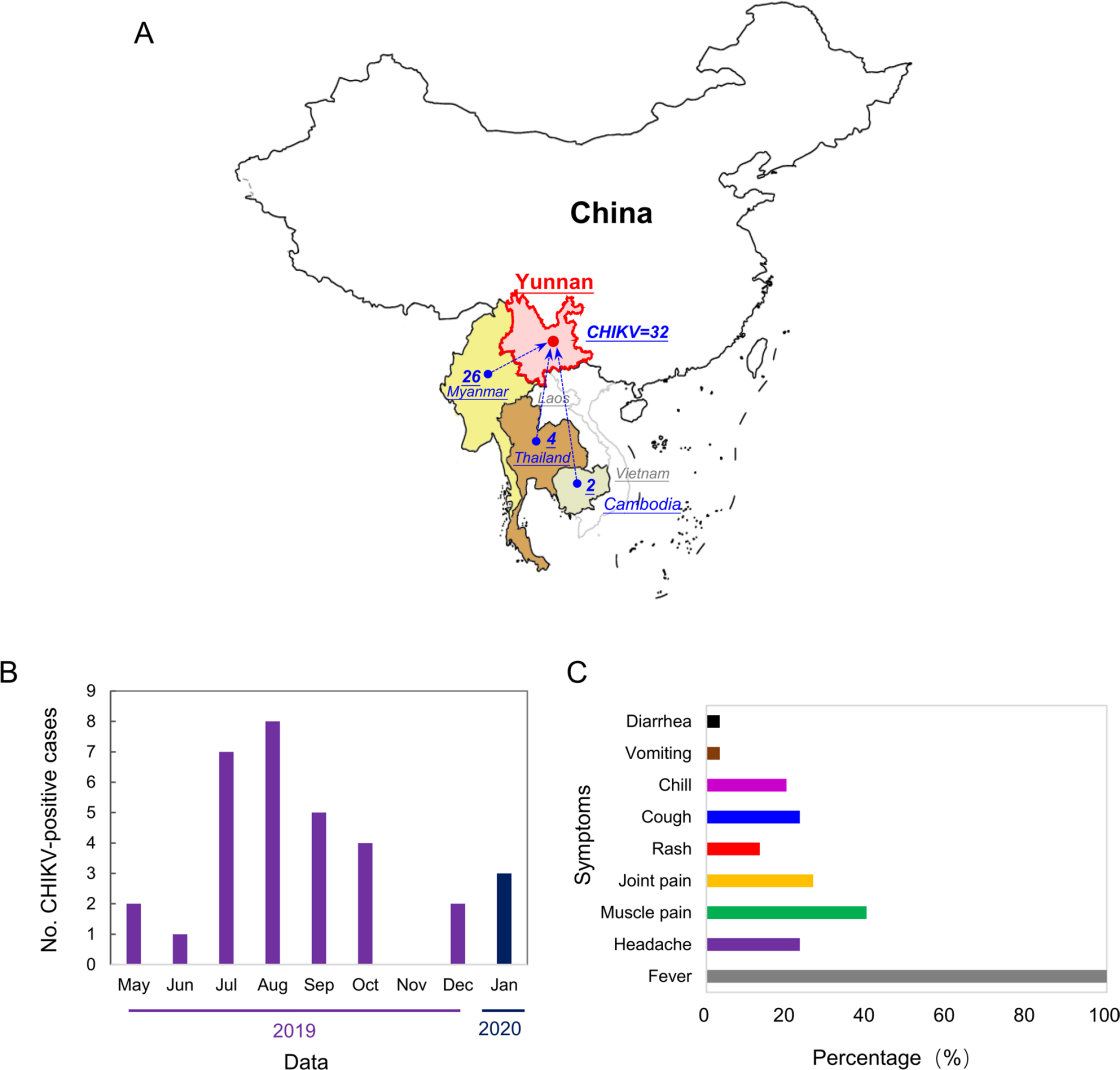
Geographical distribution, number of months, and clinical presentation of imported CHIKV infection cases in Yunnan, China, from 2019 to 2020. **A** Countries and the number of CHIKV cases imported into Yunnan from abroad. Base map source: Natural Earth (http://www.naturalearthdata.com). **B** Distribution of imported CHIKV infection cases among different months from May 2019 to January 2020. **C** Symptomatic features of imported CHIKV infection cases include joint pain, headache, cough, chills, and rash

From May 2019 to January 2020, a total of 32 cases tested positive for CHIKV RNA among 447 individuals with fever arriving at Kunming International Airport. Of these cases, 26 were imported from Myanmar, 4 from Thailand, and 2 from Cambodia (Fig. 1A). The number of cases peaked in August, when 8 cases (25%) were recorded, and then gradually decreased (Fig. 1B). Of the 32 cases, 29 (90.6%) were male and 3 (9.4%) were female. The mean age of the patients was 41.69 ± 17.97 years, with the highest number of cases in the 41–50 age group, 10 cases (31.25%). The mean body temperature of the confirmed cases was 38.8 °C ± 0.67 °C, with the highest temperature recorded being 40.1 °C in a 23-year-old student. The most commonly reported symptoms were myalgia (40.0%), followed by joint pain (26.7%), headache (23.3%), cough (23.3%), chills (20.0%), and rash (13.3%) (Fig. 1C).

### Phylogenetic and mutational analysis of CHIKV

**Fig. 2:**
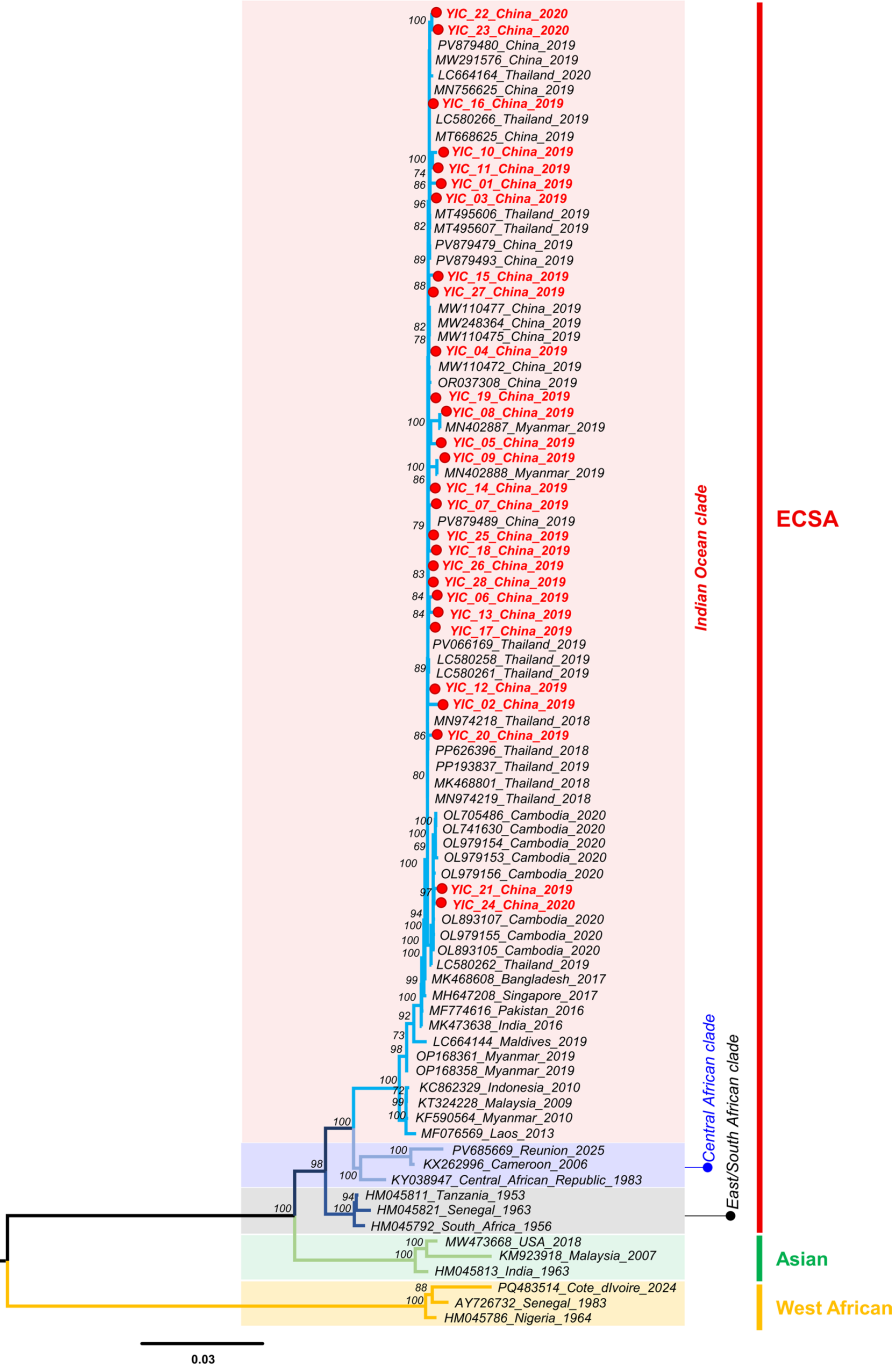
Phylogenetic analysis of Eastern/Central/Southern African (ECSA) genotypes based on complete CHIKV genome sequences. Complete sequences of representative CHIKV strains of each genotype were downloaded from the NCBI GenBank database. The red marker indicates the sample used in this study. The reference sequence for the IOL clade was identified by a BLAST search to find the best match for sequence similarity with the CHIKV strains obtained in this study

Of 32 individuals infected with CHIKV, 28 near full-length genome fragments (11,237 bp) were successfully amplified and sequenced, resulting in a success rate of 87.5% (28 of 32). The four cases that failed to amplify were likely due to low viral loads, with cycle threshold (CT) values ranging from 32.6 to 34.1. Phylogenetic analyses were performed based on the near full-length genome of CHIKV. The results showed that all 28 sequences of CHIKV isolated from the imported cases were clustered in the IOL clade of the ECSA genotype, which was highly similar to the Myanmar, Thailand, and Cambodia strains (Fig. 2).

**Fig. 3:**
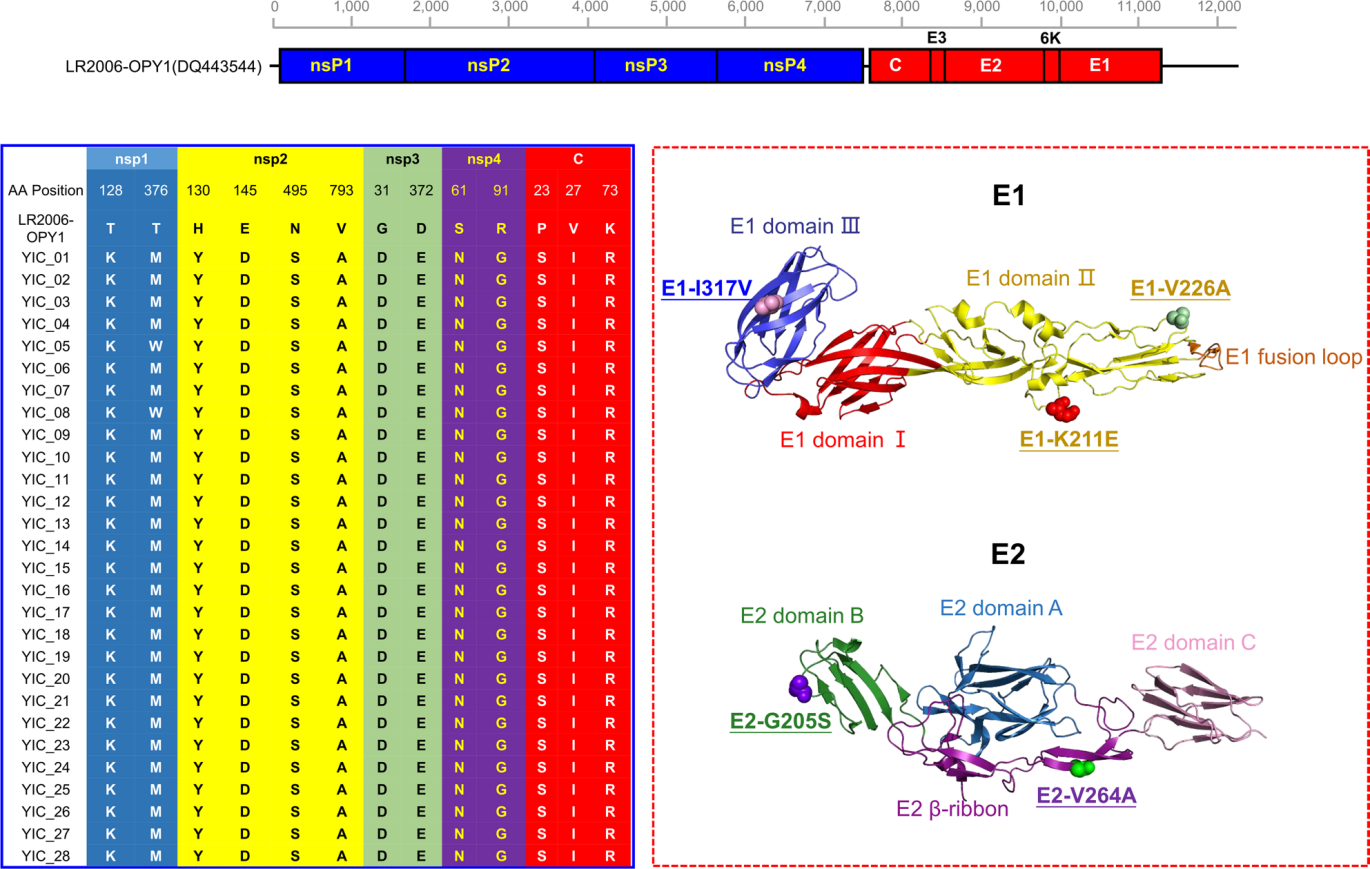
Characterization of amino acid mutations encoded by CHIKV in imported cases of infection. Amino acid comparisons of the full-length genomes were performed using a representative strain from the IOL clade (LR2006-LYP1) as a reference. Based on the structural information of CHIKV E1 and E2 found in the published literature, we searched for and downloaded the three-dimensional structure of CHIKV from the Protein Data Bank (PDB). We obtained the file in PDB format and used PyMOL software for domain analysis and visualization

To characterize the imported CHIKV genome, we performed full-length genome amino acid comparisons using a representative IOL clade strain (LR2006-LYP1) as a reference. The analysis revealed several substitutions: nsp 1 (T128K; T376M), nsp2 (H130Y; E145D; N495S; V793A), nsp3 (G31D; D372E), and C (P23S; V27I; K73R). Structural domain prediction of E1/E2 proteins using PyMOL v2.5 identified mutations in E1 domain II (K211E; V226A) and domain III (I317V), as well as in E2 domain B (G205S) and the β-ribbon region (V264A) (Fig. 3). The protein structures used for this analysis were obtained from the Protein Data Bank (PDB ID: 3N41). These results highlight key molecular variations in both non-structural and structural proteins of the imported strain.

### Diversity and distribution of CHIKV in Yunnan

**Fig. 4:**
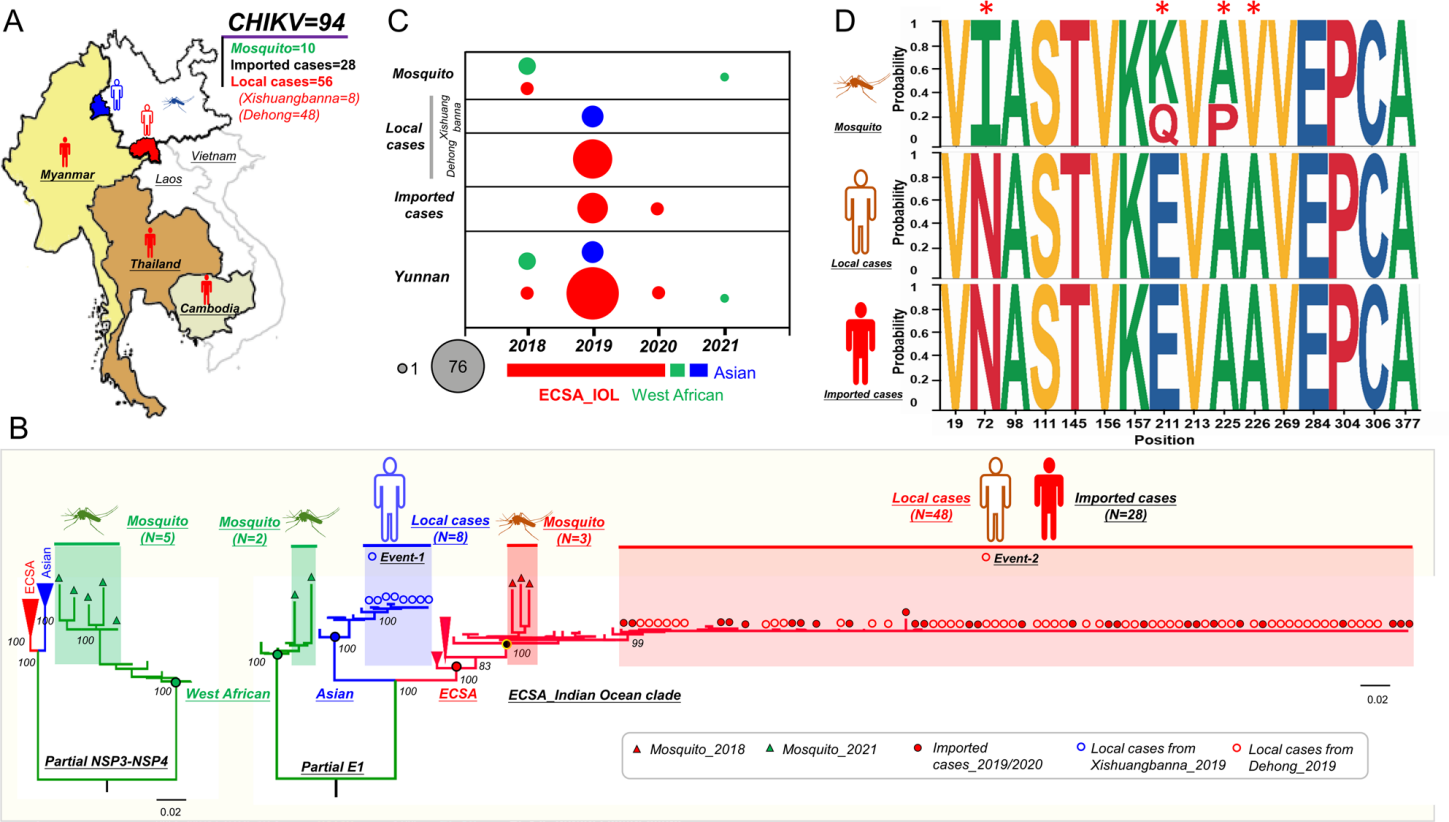
Geographical distribution of available CHIKV sequences, phylogenetic tree, genetic diversity, and mutational characteristics of CHIKV prevalent in Yunnan, China. **A** Characterization of the geographical distribution and sample sources of the 94 available CHIKV sequences identified in Yunnan from 2018 to 2021. This dataset includes 10 CHIKV sequences from Aedes aegypti, 28 cases of imported infections, and 56 cases of autochthonous transmission. Base map source: Natural Earth (http://www.naturalearthdata.com). **B** A maximum likelihood tree was constructed using partial E1 and nsp3-nsp4 gene sequences. **C** This section shows the diversity and distribution characteristics of CHIKV genotypes in Yunnan, China, from 2018 to 2024. Different colored dots represent different genotypes as indicated in the legend, with the size of the dots corresponding to the number of sequences collected. **D** Comparative analysis of E1 glycoprotein sequences identified four non-synonymous substitutions among imported cases, autochthonous cases, and mosquito strains. All the icons employed in this figure were obtained from Aigei (https://www.aigei.com/)

We conducted a comprehensive molecular epidemiology study of CHIKV in Yunnan by integrating publicly available GenBank records (*n* = 66), curating a final dataset of 94 sequences (2018–2021) stratified by origin: 10 from *Aedes aegypti*, 28 imported cases, and 56 autochthonous transmissions (Fig. 4A, Table S2). ML tree reconstruction using partial E1 and nsp3-nsp4 gene sequences showed the predominance of the IOL clade of the ECSA genotype (84.0%, 79/94), which included all imported cases, 48 autochthonous cases, and 3 mosquito isolates. Sequences of Asian genotypes (8.5%, 8/94) were exclusively derived from endemic cases, whereas West African genotypes (7.5%, 7/94) were mosquito-specific (Fig. 4B). Phylogenetic analysis revealed two distinct origins of the 2019 outbreak: Xishuangbanna’s IOL clade cluster and Dehong’s Asian genotype strains, indicating separate introduction events (Fig. 4B).

Spatiotemporal analysis revealed an epidemic concentration in 2019, with concurrent IOL-associated imported cases and two genotype-specific outbreaks. West African genotype sequences were detected in mosquito samples in 2018 and again in 2021 (Fig. 4B). A sporadic case of IOL demonstrated continued importation risk in 2020 (Fig. 4C). Comparative analysis of E1 glycoprotein sequences identified four non-synonymous substitutions that distinguish human and mosquito strains: I72V and K/Q211E, which occur together with P225A and V226A (Fig. 4D). These mutations, which are conserved in human-derived sequences regardless of genotype, suggest potential adaptive evolution facilitating anthroponotic transmission.

### Phylogenetic history of CHIKV in Yunnan

**Fig. 5:**
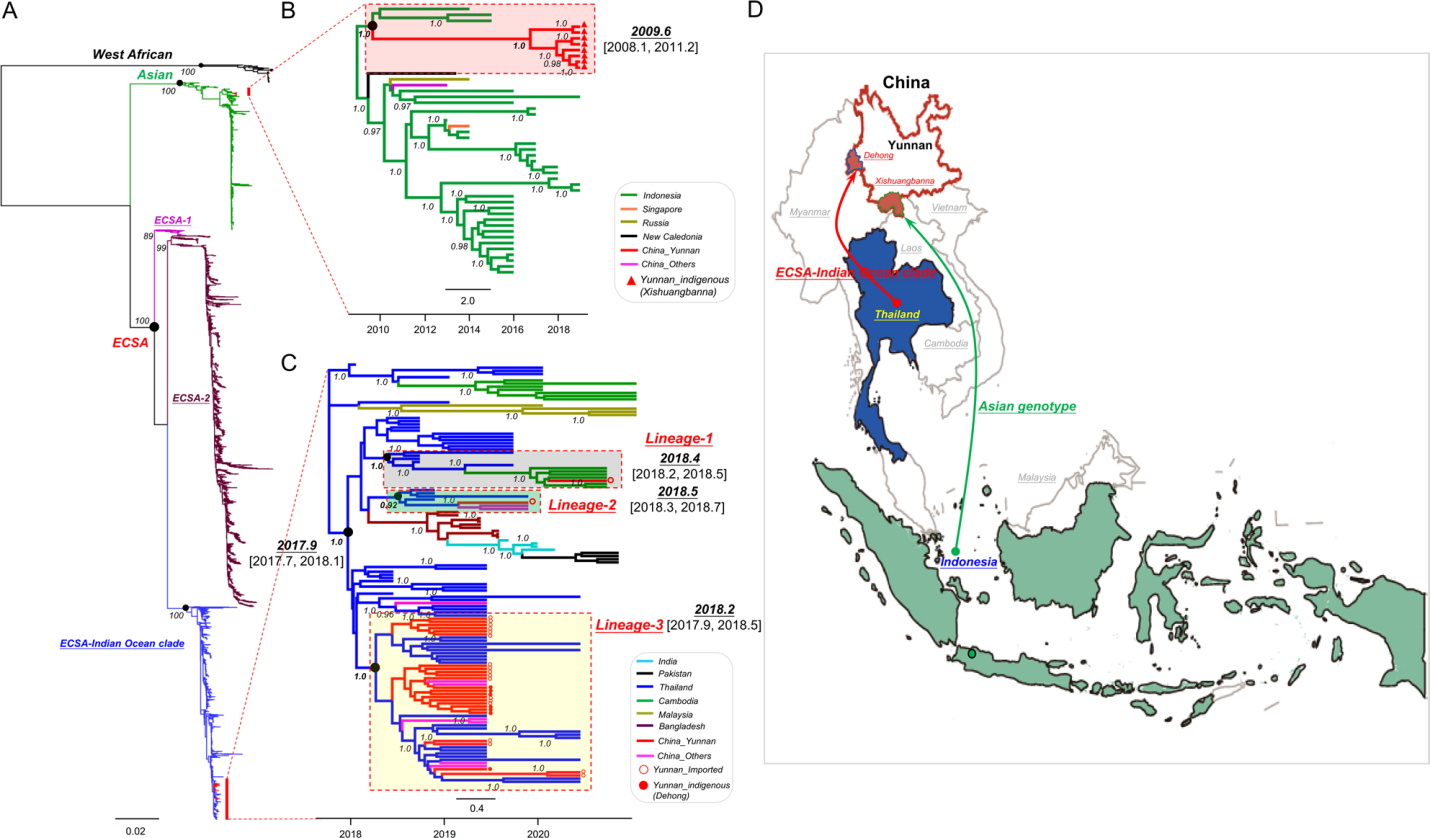
Spatio-temporal dynamics of Asian and IOL genotypes of CHIKV identified in Yunnan, China, along with the potential countries of origin for these two CHIKV genotypes. **A** The maximum likelihood tree includes sequences from the two genotypes of CHIKV (Asian = 8, IOL = 56) generated in this study, along with a total of 2,936 available full-length genome reference strains from GenBank. **B** The inset tree on the upper right represents a Maximum Clade Credibility (MCC) tree that estimates divergence times. This estimation was based on a smaller dataset (*n* = 46), which included all eight nearly complete Asian genotype sequences of CHIKV from Yunnan. Each branch of the tree is color-coded according to the legend on the right, with Yunnan sequences indicated by a red triangle. The support for the branching structure is reflected by posterior probability values ≥ 0.96. **C** The inset tree on the lower right illustrates an MCC tree that estimates divergence times. This estimation was derived from a smaller dataset (*n* = 134), which included near full-length IOL clade sequences of CHIKV from Yunnan. Each branch of the tree is color-coded according to the legend on the right, with sequences from Yunnan marked by a red circle or a solid red circle. The support for the branching structure is indicated by posterior probability values ≥ 0.92. **D**. This study identifies the potential countries of origin for the two genotypes prevalent in Yunnan, China. Base map source: Natural Earth (http://www.naturalearthdata.com)

To elucidate the phylogenetic history of CHIKV in Yunnan, we analyzed 2,979 publicly available whole-genome CHIKV sequences, comprising all available full-length genomes in GenBank as of February 2025, including 28 new isolates from this study, 15 Yunnan endemic strains, and 2,936 GenBank records. ML phylogeny showed the dominance of the IOL clade of the ECSA genotype among Yunnan strains, including both imported cases and 2019 Dehong endemic isolates [14, 15]. The secondary Asian genotype included 2019 Xishuangbanna endemic cases [16] (Fig. 5A).

We then examined the spatiotemporal dynamics of two Yunnan-endemic genotypes. We selected sequences from well-supported evolutionary clusters (bootstrap support ≥ 95%) that were most closely related to Yunnan endemic strains in the ML tree. This approach allowed us to generate two smaller datasets: one consisting of 46 Asian genotype CHIKV sequences (Fig. 5B) and the other containing 134 sequences from the IOL clade (Fig. 5C). Two significant correlations were observed between sampling date and root-to-tip genetic divergence in these datasets. For the Asian genotype dataset, the results showed an r² value of 0.849 and a correlation coefficient of 0.921 (Fig. S2). For the IOL clade dataset, the r² value was 0.805, and the correlation coefficient was 0.897 (Fig. S3). These results suggest that both datasets exhibit relatively clock-like virus evolution.

Further, phylodynamic analysis of the Yunnan endemic genotypes revealed distinct transmission patterns. For the Asian genotype, we identified a monophyletic lineage that emerged around late 2009 [95% HPD: 2008.1-2011.2], containing eight Xishuangbanna strains that clustered with Indonesian isolates, suggesting a potential epidemiological link to Indonesia (Fig. 5B). The IOL clade formed three distinct transmission lineages closely related to strains reported from Thailand: Lineage 1 (2018.4 [2018.2-2018.5]): Imported strain clustered with Cambodia and Thai isolates; Lineage 2 (2018.5 [2018.3-2018.7]): One imported case associated with Thai strains; Lineage 3 (2018.2 [2017.9-2018.5]): Dominant Dehong strains and imported isolates co-clustered with Thai variants (Fig. 5C). Notably, ancestral state reconstruction positioned sequences sampled in Thailand at basal nodes of IOL evolutionary clusters, suggesting multiple independent introductions potentially linked to Thailand (Fig. 5C). Temporal phylogeny further revealed diversification of the IOL clade in Yunnan around 2018, in contrast to the singular establishment of Asian genotypes around 2009. Taken together, the predominant CHIKV strains in Yunnan appear to be associated with two distinct transmission patterns, with Asian genotype strains clustering with Indonesian sequences and IOL clade strains clustering with Thai sequences (Fig. 5D).

## Discussion

CHIKV is divided into three main genotypes: ECSA, West African, and Asian. The ECSA genotype later gave rise to the IOL, which has caused significant epidemics and outbreaks over the past decade. In particular, IOL CHIKV was responsible for outbreaks in India, Pakistan, and Bangladesh between 2016 and 2017 [19]. In 2017, it spread from Bangladesh to Thailand, resulting in tens of thousands of suspected cases of CHIKV [19]. The IOL has also been reported in various places, including Italy in 2016–2017, Myanmar in 2019, and East African countries such as Kenya, Sudan, and Djibouti in 2014–2019 [19]. Currently, CHIKV IOL has become the most common genotype worldwide. In this study, all 28 CHIKV genotypes imported into the Yunnan region from Southeast Asian countries, including Myanmar, Thailand, and Cambodia, between May 2019 and January 2020, were of the IOL. These imported IOL CHIKV strains caused a local outbreak in the Ruili region of Yunnan, resulting in nearly 100 cases in 2019 [14, 15]. Additionally, imported CHIKV IOL cases have been reported in recent years in Guangdong, Fujian, Zhejiang, and Henan [20]. Therefore, it is imperative to strengthen surveillance and epidemiological studies on CHIKV IOLs continuously.

CHIKV is a single-stranded, positive-sense RNA virus with a genome length of 11.8 kilobases. The genome under consideration contains two open reading frames (ORFs) that code for structural and nonstructural proteins. The structural polyprotein consists of C, E3, E2, 6k, and E1 proteins, while the nonstructural proteins include nsp1, nsp2, nsp3, and nsp4. The E2 protein interacts with the E1 protein, and collectively, these proteins form three E2-E1 complexes, which constitute a trimeric envelope protein. E2 and E1 have been identified as the primary antigenic epitopes, with specific residues of E2 and E1 playing a role in receptor binding. In addition, the E1 protein has been associated with the process of membrane fusion [21]. CHIKV can increase its epidemic potential through single-nucleotide variants that can lead to clade replacement and severe outbreaks. For example, a mutation from alanine to valine at position 226 in the E1 glycoprotein (E1-A226V) of the first wave of pandemic IOL strain of CHIKV has been shown to increase midgut infectivity and facilitate viral transmission to the salivary glands and vertebrates in *Aedes albopictus* [22]. This mutation has enhanced the virus’s ability to adapt within *Aedes albopictus*, thereby conferring a selective advantage in spreading to uninfected populations [23]. As a result, an outbreak of IOL CHIKV occurred on the island of Réunion in 2005–2006, resulting in 265,000 reported clinical cases and 237 deaths [22]. The second wave of the IOL strain of CHIKV resulted in approximately 15,000 confirmed cases across 60 provinces in Thailand from 2018 to 2019 [24]. These strains exhibited the E1-K211E and E2-V264A mutations in the context of the E1-226 A mutation, resulting in increased viral titers in the midgut, saliva, legs, and wings of *Aedes aegypti* following infection [25]. In this study, we identified 28 imported cases of CHIKV, all belonging to the IOL strain, from the second wave of the epidemic. Our strains are characterized by the notable mutations E1-K211E and E2-V264A. When we compared these strains with a representative IOL strain from the first wave of the epidemic, designated LR2006, we discovered several potentially significant amino acid mutation sites in the identified strains. In particular, the nsp1-T376M mutation is located near the nsp1-nsp4 interface and could affect how nsp4 docks to the oligomeric ring of nsp1 [26]. The nsp2-A793V mutation introduces a larger hydrophobic group on the surface of the short, disordered C-terminal peptide of the nsp2 protease domain, which is unlikely to confer any advantage to the virus [26]. Additionally, the E2-G205S mutation may serve as an immune evasion mechanism, potentially enhancing viral fitness [22, 26]. However, further studies in mosquito vectors and mice are needed to determine whether these mutations can enhance CHIKV transmission, adaptation, and pathogenicity.

Our results indicate that both the imported CHIKV strains in Yunnan in 2019 and the locally circulating strains from Dehong (Ruili) belong to the IOL, forming a tight cluster. This supports previous conclusions that the 2019 outbreak in Dehong was caused by imported cases [14, 15]. In contrast, sequences from the 2019 outbreak in Xishuangbanna clustered within the Asian genotype [16]. This suggests that two distinct CHIKV genotypes, IOL and Asian, co-circulated in separate regions of Yunnan in the same year, likely due to independent epidemiological events. Notably, recent studies have also identified novel West African genotypes and IOL strains in *Aedes aegypti* populations in Yunnan, with successful virus isolation from mosquitoes [27]. This suggests that a potential third CHIKV genotype may be establishing local transmission cycles, adding to the complexity of CHIKV genotype diversity in the region. Taken together, these findings pose significant challenges for the prevention and control of chikungunya in Yunnan, China.

Further, our molecular epidemiological investigations reveal two distinct pathways for the introduction of CHIKV into Yunnan Province. For the ECSA genotype IOL clade, Bayesian time-scaled phylogenies show that strains from the 2019 Ruili outbreak, along with imported cases linked to Myanmar, Thailand, and Cambodia, form a monophyletic cluster with sequences sampled in Thailand (posterior probability = 1.0) and diverged around 2017–2018 (Fig. 5C). This timing is consistent with the resurgence of IOL-CHIKV in Thailand (2016–2019), which resulted in more than 18,000 confirmed cases, mainly concentrated in the northern province of Chiang Mai, near the Myanmar border, where seroprevalence in febrile patients reached 34.5% [26, 28]. In addition, three lines of evidence suggest a potential epidemiological link between Yunnan IOL strains and those reported from Thailand. First, in addition to the strain prevalent in Yunnan, genetically similar strains have spread to Laos and Cambodia, largely due to cross-border labor migration [29–31]. Second, mutations such as E1-K211E and E2-V264A, which increase infectivity, spread, and transmission in *Aedes aegypti*, have been conserved in Thai strains since 2018. This facilitates the establishment of CHIKV in *Aedes aegypti* ecosystems in Yunnan [26]. Third, trade between Yunnan and Thailand increased by 53.1% in 2019, reaching $1.581 billion [32]. This increase in trade was accompanied by significant population movement, with Yunnan receiving 554,700 Thai tourists and 10.98 million Chinese tourists visiting Thailand, an increase of 4.2% over the previous year [33].

In contrast, the Asian genotype of CHIKV responsible for the 2019 outbreak in Xishuangbanna clustered with Indonesian lineages that have been circulating since about 2007, as indicated by phylogenetic clustering with Indonesian isolates (posterior probability = 0.93) (Fig. 5). This correlates with the CHIKV epidemics in Indonesia from 2005 to 2013, where more than 200,000 cases were reported. In addition, the genotypes of the outbreaks in Indonesia before 2008 were all Asian [34]. Since the establishment of the strategic partnership between China and Indonesia in 2005, trade and exchanges between the two countries have accelerated, especially in Yunnan, due to the opening of new routes such as Kunming-Jakarta-Bali and Kunming-Medan-Jakarta [35].

This study has several limitations. First, our analysis focused only on imported fever cases from Kunming Airport in 2019, excluding cross-border fever cases from other border ports in Yunnan. This geographic restriction may underrepresent the true genetic diversity of CHIKV circulating in Yunnan, as border regions such as Ruili or Lincang, frequent hotspots for arboviral importation, were not sampled. As a result, the prevalence and lineage distribution of CHIKV in these areas remain unclear. Second, relying on RT-qPCR for CHIKV detection without integrating serologic assays (e.g., IgM/IgG testing) limits our ability to identify past infections or confirm acute-phase cases with low viral loads. Third, although we analyzed all available Yunnan CHIKV sequences from GenBank, the dataset consisted predominantly of partial E1 gene sequences from Dehong and Xishuangbanna. This introduces geographic and genetic biases, as CHIKV whole-genome data are critical for robust phylogenetic reconstruction, and sparse sampling from other prefectures may mask emerging variants or localized transmission networks. Future studies should expand surveillance to multiple border ports, combine molecular and serological diagnostics, and prioritize whole-genome sequencing to comprehensively elucidate CHIKV epidemiology in this high-risk region.

## Conclusions

In summary, the present study investigated the epidemiology and genetic diversity of CHIKV samples imported from Southeast Asia to Yunnan, China, between 2019 and 2020. The identification of all strains as part of the IOL of the ECSA genotype was determined. It is noteworthy that these strains exhibited mutations E1-211E, E1-226 A, and E2-264 A, which have been demonstrated to enhance transmission by *Aedes albopictus* mosquitoes. This finding underscores Yunnan as a significant area for cross-border CHIKV transmission and highlights the urgent need for improved border screening, genomic surveillance, and targeted vector control strategies.

## Supplementary Information

Supplementary Material 1. Table S1: Primers used for CHIKV genome amplification and sequencing. Table S2: Sequence information from CHIKV circulating in Yunnan, China, available in the Genbank database.

Supplementary Material 2. Fig. S1: Schematic representation of the overlapping amplicons covering the full-length CHIKV genome. Fig. S2: Temporal signal analysis of the Asian lineage dataset was performed in TempEst. Fig. S3: Temporal signal analysis of the ECSA–Indian Ocean clade dataset performed in TempEst.

## Data Availability

All relevant data are within the manuscript and its Supporting Information files. The nucleotide sequences generated in this study have been submitted to GenBank with accession numbers MN402883-MN402892 and PV022110-PV022127. The datasets supporting the findings of this study are publicly available at GitHub: https://github.com/weichang292/CHIKV-Yunnan-data.

## Abbreviations

CHIKV: Chikungunya virus
CT: Cycle Threshold
ECSA: East/Central/South African
EDTA: Ethylenediaminetetraacetic acid
EMA: European Medicines Agency
FDA: Food and Drug Administration
GTR: General Time Reversible
HPD: Highest Posterior Density
IOL: Indian Ocean lineage
MCC: Maximum Clade Credibility
MCMC: Markov Chain Monte Carlo
ML: Maximum Likelihood
nsp: Non-structural protein
ORFs: Open Reading Frames
RT: Reverse Transcription
