# Supplementary material for: Genomic surveillance reveals multiple origins and local transmission of travel-associated chikungunya virus in Yunnan, China
Source: BMC Microbiol. 2026 Apr 25;26:541. doi: 10.1186/s12866-026-05100-w (PMC13248379; doi:10.1186/s12866-026-05100-w)
Supplement: Supplementary file 1 — Supplementary Material 1. Table S1: Primers used for CHIKV genome amplification and sequencing. Table S2: Sequence information from CHIKV circulating in Yunnan, China, available in the Genbank database. [file 12866_2026_5100_MOESM1_ESM.docx]

**Table S1.** **Primers used for CHIKV genome amplification and sequencing**

| **Fragment** | **Primers** | **Sequences（5’-3’）** | **Binding sites** | **Length of PCR products** |
| --- | --- | --- | --- | --- |
| F1 | 1CHIKV01F | CTCTGCAAAGCAAGAGATTAATAACCCATC | 46-76 | 1297bp |
|  | 1CHIKV01R | ATGCCCATAGACAGCAGCAGGTCAG | 1319-1343 |  |
|  | 1CHIKV02F* | GAGATTAATAACCCATCATGGATCCTGTGTA | 60-90 | 1228bp |
|  | 1CHIKV02R* | TCTTCCATGTCTTTCCGGCACTCCT | 1263-1287 |  |
| F2 | 2CHIKV01F | TACAGAAGTCACGCCGGAGGATGC | 1117-1140 | 1286bp |
|  | 2CHIKV01R | CATTCAAGAGCAGCGAGTCAACCGTA | 2377-2402 |  |
|  | 2CHIKV02F* | CAGAGAATAGTGGTTAACGGCAGAACG | 1166-1192 | 1126bp |
|  | 2CHIKV02R* | CCTGCCTGGTAACTAGGTTCTTGATAATAG | 2262-2291 |  |
| F3 | 3CHIKV01F | AAGAGTCGTATGAGCTGGTGAGGGCAG | 2031-2057 | 1220bp |
|  | 3CHIKV01R | CGTACATATTTCATTCAGGGCTACTTCAGG | 3221-3250 |  |
|  | 3CHIKV02F* | GTCTACGACGTGGATCAGAGAAGATGC | 2078-2104 | 1015bp |
|  | 3CHIKV02R* | ATGTATCGAAGGTCATTTGGTGACTGC | 3066-3092 |  |
| F4 | 4CHIKV01F | CGTCAGAGCACGTCAACGTACTCCTAA | 2898-2924 | 1339bp |
|  | 4CHIKV01R | GTTCCCACTGGTGTTGCACTGTTCTT | 4211-4236 |  |
|  | 4CHIKV02F* | CTGCAGAACCCACCGAAAGGAAAC | 2984-3007 | 1153bp |
|  | 4CHIKV02R* | TGACTACGCACTCTTCATCGTTCTTCG | 4110-4136 |  |
| F5 | 5CHIKV01F | CGTCTAGAGCGTTGAAACCACCAT | 3921-3944 | 1273bp |
|  | 5CHIKV01R | TCGTCTAGTGCTGGTTCTAGGGCTG | 5169-5193 |  |
|  | 5CHIKV02F* | TTCAGCAACTTTGACAATGGCAGAAG | 3977-4002 | 1175bp |
|  | 5CHIKV02R* | TCTGACGGGACGGGCAGTATCT | 5130-5151 |  |
| F6 | 6CHIKV01F | GCTATTTGACCATAACGTGCCATCGC | 5002-5027 | 1192bp |
|  | 6CHIKV01R | GTGCTGTTTCGGGTAGCTCCTGAGTT | 6168-6193 |  |
|  | 6CHIKV02F* | GCGCGTAAGTCCAAGGGAATATAGAT | 5026-5048 | 1112bp |
|  | 6CHIKV02R* | AACTCTCCGACCCGTCCACCAT | 6116-6137 |  |
| F7 | 7CHIKV01F | ATTGTCCAATCCCGAGTCCGCAGT | 6007-6030 | 1165bp |
|  | 7CHIKV01R | GGCTTTCAAGGATACAACTGCATCTATGA | 7143-7171 |  |
|  | 7CHIKV02F* | CAGCATGCAATGAGTTCTTAGCCAGA | 6033-6058 | 1076bp |
|  | 7CHIKV02R* | GGCTGCCATCAATTCATCGGAGAC | 7085-7108 |  |
| F8 | 8CHIKV01F | TACCGACAGGTACGCGCTTCAAGTT | 6912-6936 | 1250bp |
|  | 8CHIKV01R | CTCCTGAGTACTGTACTGCTCCGTGGT | 8135-8161 |  |
|  | 8CHIKV02F* | CTCTGTTCGTCAACACATTGTTAAACAT | 6969-6996 | 1149bp |
|  | 8CHIKV02R* | TCCGGTTTCTCATGGGTGAACTTC | 8094-8117 |  |
| F9 | 9CHIKV01F | GTCAAGCACGAAGGTAAGGTAACAGGTT | 7915-7942 | 1236bp |
|  | 9CHIKV01R | GCAATTACACTTATACCGCACCGTCTG | 9124-9150 |  |
|  | 9CHIKV02F* | GGGGACAAAGTAATGAAACCAGCACAT | 7957-7983 | 1152bp |
|  | 9CHIKV02R* | CTTTACGTTGCCGGACTGTTGTGAC | 9084-9108 |  |
| F10 | 10CHIKV01F | TACGCACCCATTTCACCACGACC | 8916-8938 | 1193bp |
|  | 10CHIKV01R | CCAAAGTGACTGACAGTAGTTCCATCTCC | 10080-10108 |  |
|  | 10CHIKV02F* | AAATTCCATTCCCGACCGCAGC | 8959-8980 | 1104bp |
|  | 10CHIKV02R* | GCCAGGTCTATTGACTAGAGTCTTATACGG | 10033-10062 |  |
| F11 | 11CHIKV01F | GATATACCTGTGGAACGAGCAGCAACC | 9831-9857 | 1338bp |
|  | 11CHIKV01R | GCCGGGTAGTTGACTATGTGGTCCTT | 11143-11168 |  |
|  | 11CHIKV02F* | CGCTGGCAGCCCTGATTGTTCTAT | 9884-9907 | 1252bp |
|  | 11CHIKV02R* | TGGCACTCAGCTGCACAGTGTACTTG | 11110-11135 |  |
| F12 | 12CHIKV01F | TGTGCGGTGCATTCGATGACTAAC | 10975-10998 | 822bp |
|  | 12CHIKV01R | AAATAACATCTCCTACGTCCCTGTGG | 11771-11796 |  |
|  | 12CHIKV02F* | GGGAAGCTGAGATAGAAGTTGAAGGGAATTC | 11012-11042 | 747bp |
|  | 12CHIKV02R* | GGGAAGCTGAGATAGAAGTTGAAGGGAATTC | 11728-11758 |  |

* Indicates inner primers and sequencing primers.

**Table S2. Sequence information from CHIKV circulating in Yunnan, China available in Genbank database**

| **Accession** | **Organism_Name** | **Nuc_Location** | | **Host** | **Collection_Date** | **Geo_Location** | **Source** | **Genotype** |
| --- | --- | --- | --- | --- | --- | --- | --- | --- |
| OK316989^1^ | IMBCAMS-1156 | Full-length | | Homo sapiens | 2019 | Xishuangbanna | Autochthonous | Asian |
| OK316990^1^ | IMBCAMS-1156 | Full-length | | Homo sapiens | 2019 | Xishuangbanna | Autochthonous | Asian |
| OK316991^1^ | IMBCAMS-1151 | Full-length | | Homo sapiens | 2019 | Xishuangbanna | Autochthonous | Asian |
| OK316992^1^ | IMBCAMS-1151 | Full-length | | Homo sapiens | 2019 | Xishuangbanna | Autochthonous | Asian |
| OK316993^1^ | IMBCAMS-631 | Full-length | | Homo sapiens | 2019 | Xishuangbanna | Autochthonous | Asian |
| OK316994^1^ | IMBCAMS-631 | Full-length | | Homo sapiens | 2019 | Xishuangbanna | Autochthonous | Asian |
| OK316995^1^ | IMBCAMS-625 | Full-length | | Homo sapiens | 2019 | Xishuangbanna | Autochthonous | Asian |
| OK316996^1^ | IMBCAMS-625 | Full-length | | Homo sapiens | 2019 | Xishuangbanna | Autochthonous | Asian |
| PP501552^2^ | CHIKV2019_01 | Full-length | | Homo sapiens | 2019 | Dehong (Ruili) | Autochthonous | ECSA_IOL |
| PP501554^2^ | CHIKV2019_02 | Full-length | | Homo sapiens | 2019 | Dehong (Ruili) | Autochthonous | ECSA_IOL |
| MW248363 | YNRL1 2019 | Full-length | | Homo sapiens | 2019 | Dehong (Ruili) | Autochthonous | ECSA_IOL |
| MW248364 | YNRL2 2019 | Full-length | | Homo sapiens | 2019 | Dehong (Ruili) | Autochthonous | ECSA_IOL |
| MW291576 | YNRL3 2019 | Full-length | | Homo sapiens | 2019 | Dehong (Ruili) | Autochthonous | ECSA_IOL |
| MW110472^3^ | 19RL05(2019) | Full-length | | Homo sapiens | 2019 | Dehong (Ruili) | Autochthonous | ECSA_IOL |
| MW110473^3^ | 19RL33(2019) | Full-length | | Homo sapiens | 2019 | Dehong (Ruili) | Autochthonous | ECSA_IOL |
| MW110474^3^ | 19RL34(2019) | Full-length | | Homo sapiens | 2019 | Dehong (Ruili) | Autochthonous | ECSA_IOL |
| MW110475^3^ | 19RL45(2019) | Full-length | | Homo sapiens | 2019 | Dehong (Ruili) | Autochthonous | ECSA_IOL |
| MW110476^3^ | 19RL50(2019) | Full-length | | Homo sapiens | 2019 | Dehong (Ruili) | Autochthonous | ECSA_IOL |
| MW110477^3^ | 19RL51(2019) | Full-length | | Homo sapiens | 2019 | Dehong (Ruili) | Autochthonous | ECSA_IOL |
| MN747049^3^ | 19RL04(2019) | 10004-11323 | | Homo sapiens | 2019 | Dehong (Ruili) | Autochthonous | ECSA_IOL |
| MN747050^3^ | 19RL05(2019) | 10004-11323 | | Homo sapiens | 2019 | Dehong (Ruili) | Autochthonous | ECSA_IOL |
| MN747051^3^ | 19RL07(2019) | 10004-11323 | | Homo sapiens | 2019 | Dehong (Ruili) | Autochthonous | ECSA_IOL |
| MN747052^3^ | 19RL09(2019) | 10004-11323 | | Homo sapiens | 2019 | Dehong (Ruili) | Autochthonous | ECSA_IOL |
| MN747053^3^ | 19RL11(2019) | 10004-11323 | | Homo sapiens | 2019 | Dehong (Ruili) | Autochthonous | ECSA_IOL |
| MN747054^3^ | 19RL13(2019) | 10004-11323 | | Homo sapiens | 2019 | Dehong (Ruili) | Autochthonous | ECSA_IOL |
| MN747055^3^ | 19RL15(2019) | 10004-11323 | | Homo sapiens | 2019 | Dehong (Ruili) | Autochthonous | ECSA_IOL |
| MN747056^3^ | 19RL17(2019) | 10004-11323 | | Homo sapiens | 2019 | Dehong (Ruili) | Autochthonous | ECSA_IOL |
| MN747057^3^ | 19RL20(2019) | 10004-11323 | | Homo sapiens | 2019 | Dehong (Ruili) | Autochthonous | ECSA_IOL |
| MN747058^3^ | 19RL21(2019) | 10004-11323 | | Homo sapiens | 2019 | Dehong (Ruili) | Autochthonous | ECSA_IOL |
| MN747059^3^ | 19RL22(2019) | 10004-11323 | | Homo sapiens | 2019 | Dehong (Ruili) | Autochthonous | ECSA_IOL |
| MN747060^3^ | 19RL23(2019) | 10004-11323 | | Homo sapiens | 2019 | Dehong (Ruili) | Autochthonous | ECSA_IOL |
| MN747061^3^ | 19RL24(2019) | 10004-11323 | | Homo sapiens | 2019 | Dehong (Ruili) | Autochthonous | ECSA_IOL |
| MN747062^3^ | 19RL25(2019) | 10004-11323 | | Homo sapiens | 2019 | Dehong (Ruili) | Autochthonous | ECSA_IOL |
| MN747063^3^ | 19RL27(2019) | 10004-11323 | | Homo sapiens | 2019 | Dehong (Ruili) | Autochthonous | ECSA_IOL |
| MN747064^3^ | 19RL29(2019) | 10004-11323 | | Homo sapiens | 2019 | Dehong (Ruili) | Autochthonous | ECSA_IOL |
| MN747065^3^ | 19RL30(2019) | 10004-11323 | | Homo sapiens | 2019 | Dehong (Ruili) | Autochthonous | ECSA_IOL |
| MN747066^3^ | 19RL32(2019) | 10004-11323 | | Homo sapiens | 2019 | Dehong (Ruili) | Autochthonous | ECSA_IOL |
| MN747067^3^ | 19RL33(2019) | 10004-11323 | | Homo sapiens | 2019 | Dehong (Ruili) | Autochthonous | ECSA_IOL |
| MN747068^3^ | 19RL34(2019) | 10004-11323 | | Homo sapiens | 2019 | Dehong (Ruili) | Autochthonous | ECSA_IOL |
| MN747069^3^ | 19RL37(2019) | 10004-11323 | | Homo sapiens | 2019 | Dehong (Ruili) | Autochthonous | ECSA_IOL |
| MN747070^3^ | 19RL38(2019) | 10004-11323 | | Homo sapiens | 2019 | Dehong (Ruili) | Autochthonous | ECSA_IOL |
| MN747071^3^ | 19RL39(2019) | 10004-11323 | | Homo sapiens | 2019 | Dehong (Ruili) | Autochthonous | ECSA_IOL |
| MN747072^3^ | 19RL40(2019) | 10004-11323 | | Homo sapiens | 2019 | Dehong (Ruili) | Autochthonous | ECSA_IOL |
| MN747073^3^ | 19RL41(2019) | 10004-11323 | | Homo sapiens | 2019 | Dehong (Ruili) | Autochthonous | ECSA_IOL |
| MN747074^3^ | 19RL42(2019) | 10004-11323 | | Homo sapiens | 2019 | Dehong (Ruili) | Autochthonous | ECSA_IOL |
| MN747075^3^ | 19RL43(2019) | 10004-11323 | | Homo sapiens | 2019 | Dehong (Ruili) | Autochthonous | ECSA_IOL |
| MN747076^3^ | 19RL44(2019) | 10004-11323 | | Homo sapiens | 2019 | Dehong (Ruili) | Autochthonous | ECSA_IOL |
| MN747077^3^ | 19RL45(2019) | 10004-11323 | | Homo sapiens | 2019 | Dehong (Ruili) | Autochthonous | ECSA_IOL |
| MN747078^3^ | 19RL46(2019) | 10004-11323 | | Homo sapiens | 2019 | Dehong (Ruili) | Autochthonous | ECSA_IOL |
| MN747079^3^ | 19RL47(2019) | 10004-11323 | | Homo sapiens | 2019 | Dehong (Ruili) | Autochthonous | ECSA_IOL |
| MN747080^3^ | 19RL48(2019) | 10004-11323 | | Homo sapiens | 2019 | Dehong (Ruili) | Autochthonous | ECSA_IOL |
| MN747081^3^ | 19RL49(2019) | 10004-11323 | | Homo sapiens | 2019 | Dehong (Ruili) | Autochthonous | ECSA_IOL |
| MN747082^3^ | 19RL50(2019) | 10004-11323 | | Homo sapiens | 2019 | Dehong (Ruili) | Autochthonous | ECSA_IOL |
| MN747083^3^ | 19RL51(2019) | 10004-11323 | | Homo sapiens | 2019 | Dehong (Ruili) | Autochthonous | ECSA_IOL |
| MN747084^3^ | 19RL53(2019) | 10004-11323 | | Homo sapiens | 2019 | Dehong (Ruili) | Autochthonous | ECSA_IOL |
| MN747085^3^ | 19RL54(2019) | 10004-11323 | | Homo sapiens | 2019 | Dehong (Ruili) | Autochthonous | ECSA_IOL |
| OQ054327 | CHIKV-China/YN2021-1 | Full-length | | Mosquito | 2021 | Yunnan | Autochthonous | West Africa |
| MZ494489^4^ | CHIKV-China/YN2018E1-1 | 10004-11323 | | Mosquito | 2018 | Yunnan | Autochthonous | West African |
| MZ494493^4^ | CHIKV-China/YN2018NS3-1 | 5678-6745 | | Mosquito | 2018 | Yunnan | Autochthonous | West African |
| MZ494494^4^ | CHIKV-China/YN2018NS3-2 | 5678-6745 | | Mosquito | 2018 | Yunnan | Autochthonous | West African |
| MZ494495^4^ | CHIKV-China/YN2018NS3-3 | 5678-6745 | | Mosquito | 2018 | Yunnan | Autochthonous | West African |
| MZ494496^4^ | CHIKV-China/YN2018NS3-4 | 5678-6745 | | Mosquito | 2018 | Yunnan | Autochthonous | West African |
| MZ494497^4^ | CHIKV-China/YN2018NS3-5 | 5678-6745 | | Mosquito | 2018 | Yunnan | Autochthonous | West African |
| MZ494490^4^ | CHIKV-China/YN2018E1-2 | 10004-11323 | | Mosquito | 2018 | Yunnan | Autochthonous | ECSA_IOL |
| MZ494491^4^ | CHIKV-China/YN2018E1-3 | 10004-11323 | | Mosquito | 2018 | Yunnan | Autochthonous | ECSA_IOL |
| MZ494492^4^ | CHIKV-China/YN2018E1-4 | | 10004-11323 | Mosquito | 2018 | Yunnan | Autochthonous | ECSA_IOL |

1. Zou M, Su C, Li T, Zhang J, Li D, Luan N, et al. Genetic Characterization of Chikungunya Virus Among Febrile Dengue Fever-Like Patients in Xishuangbanna, Southwestern Part of China. Front Cell Infect Microbiol. 2022;12:914289.

2. Xie L, Wu Y, Jiang J, Zhou H. An improved alphaviruses-specific RT-qPCR facilitates monitoring and prevention of alphaviruses. J Med Virol. 2024;96(7):e29788.

3. Yin X, Hu TS, Zhang H, Liu Y, Zhou Z, Liu L, et al. Emergent chikungunya fever and vertical transmission in Yunnan Province, China, 2019. Arch Virol. 2021;166(5):1455-1462.

4. Feng G, Zhang J, Zhang Y, Li C, Zhang D, Li Y, et al. Metagenomic Analysis of Togaviridae in Mosquito Viromes Isolated From Yunnan Province in China Reveals Genes from Chikungunya and Ross River Viruses. Front Cell Infect Microbiol. 2022;12:849662.
