# Supplementary material for: Genomic surveillance reveals multiple origins and local transmission of travel-associated chikungunya virus in Yunnan, China
Source: BMC Microbiol. 2026 Apr 25;26:541. doi: 10.1186/s12866-026-05100-w (PMC13248379; doi:10.1186/s12866-026-05100-w)
Supplement: Supplementary file 2 — Supplementary Material 2. Fig. S1: Schematic representation of the overlapping amplicons covering the full-length CHIKV genome. Fig. S2: Temporal signal analysis of the Asian lineage dataset was performed in TempEst. Fig. S3: Temporal signal analysis of the ECSA–Indian Ocean clade dataset performed in TempEst. [file 12866_2026_5100_MOESM2_ESM.pdf]

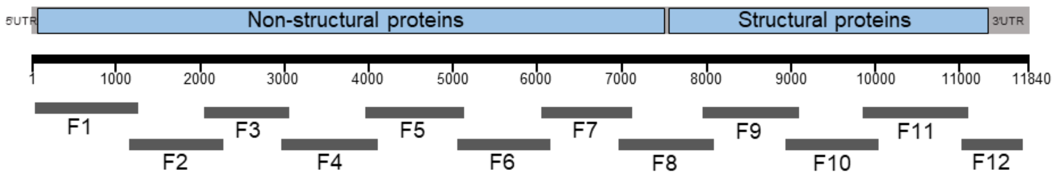

Fig S1. Schematic representation of the overlapping amplicons covering the full-length CHIKV genome. The genome was amplified using 12 overlapping fragments (F1–F12). The positions of each fragment correspond to the primer pairs listed in Table S1.

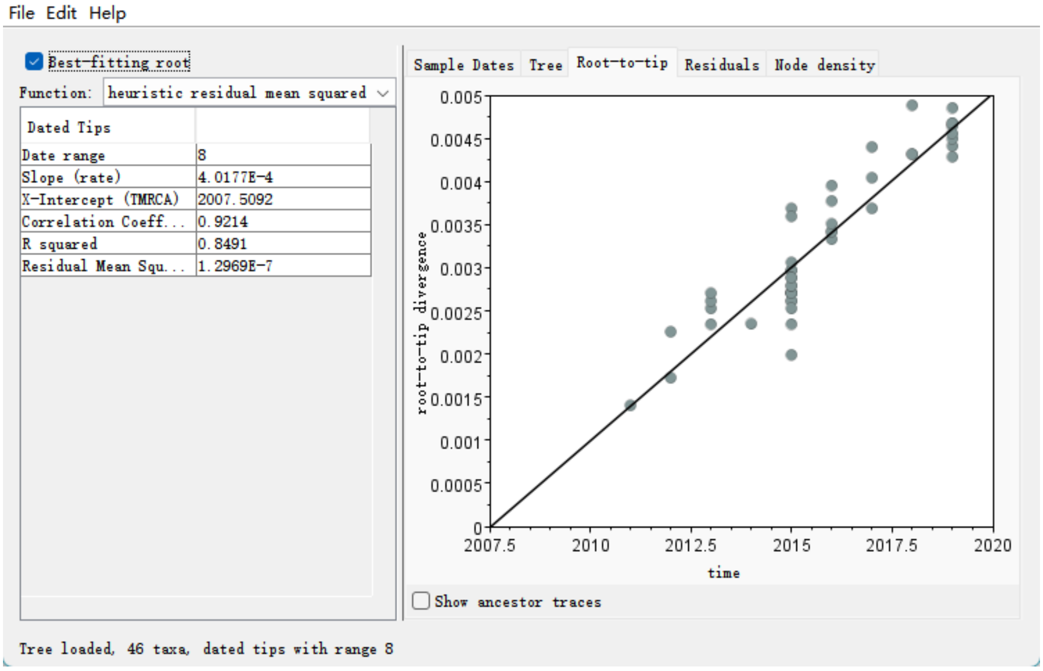

Fig. S2 Temporal signal analysis of the *Asian* lineage dataset was performed in TempEst. A regression of root-to-tip genetic distance against sampling year was conducted using the maximum likelihood tree generated by FastTree. The analysis revealed a strong temporal signal, with a correlation coefficient of 0.921 and an $R^2 = 0.849$, indicating that the dataset is suitable for molecular clock analysis. The estimated evolutionary rate (slope) was 4.02×10^{-4} substitutions/site/year, and the best-fitting root corresponded to an intercept year of 2007.51.

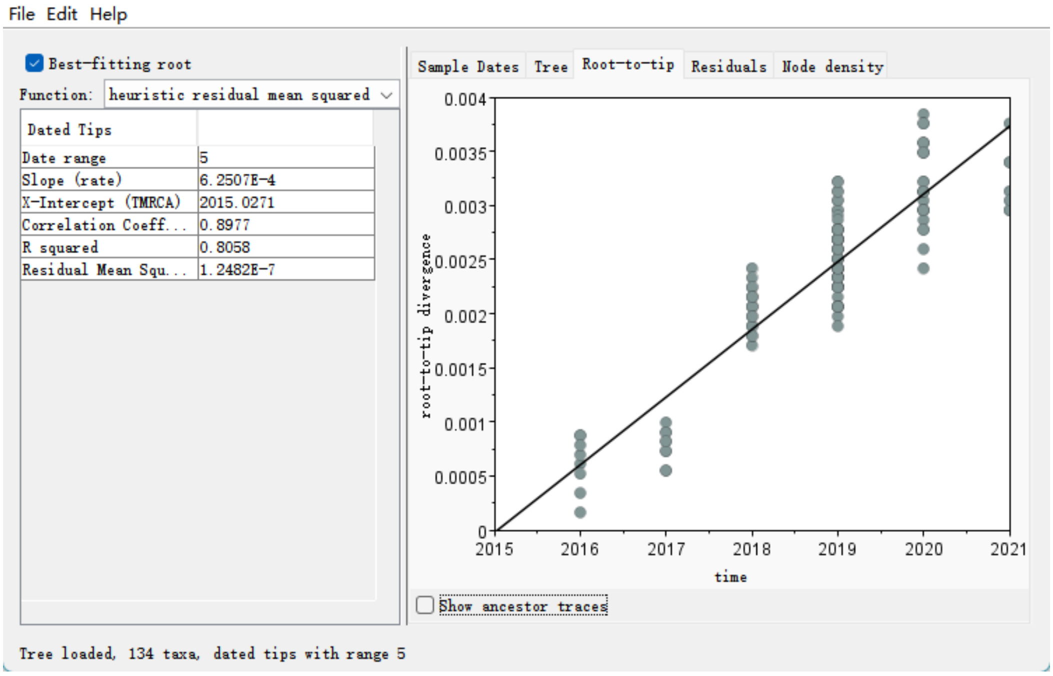

Fig. S3 Temporal signal analysis of the *ECSA–Indian Ocean clade* dataset performed in TempEst. A regression of root-to-tip genetic distances against sampling times was conducted using the maximum-likelihood tree inferred by FastTree. The dataset exhibited a strong temporal signal, with a correlation coefficient of 0.897 and $R^2 = 0.805$, indicating sufficient clock-like structure for molecular clock analysis. The best-fitting root was determined heuristically, yielding an estimated evolutionary rate (slope) of 6.25×10^{-4} substitutions/site/year and an intercept year of 2015.03.
